# Engineering Natural and Recombinant Silks for Sustainable Biodevices

**DOI:** 10.3389/fchem.2022.881028

**Published:** 2022-05-05

**Authors:** Xinchen Shen, Haoyuan Shi, Hongda Wei, Boxuan Wu, Qingyuan Xia, Jingjie Yeo, Wenwen Huang

**Affiliations:** ^1^ The Zhejiang University - University of Edinburgh Institute, Zhejiang University School of Medicine, Zhejiang University, Hangzhou, China; ^2^ J^2^ Lab for Engineering Living Materials, Sibley School of Mechanical and Aerospace Engineering, Cornell University, Ithaca, NY, United States; ^3^ Dr. Li Dak Sum and Yip Yio Chin Center for Stem Cells and Regenerative Medicine and Department of Orthopedics of the Second Affiliated Hospital, Zhejiang University School of Medicine, Zhejiang University, Hangzhou, China

**Keywords:** silk, recombinant protein, micro-nano fabrication technology, multiscale simulation, biodevices

## Abstract

Silk fibroin (SF) is a structural protein derived from natural silkworm silks. Materials fabricated based on SF usually inherit extraordinary physical and biological properties, including high mechanical strength, toughness, optical transparency, tailorable biodegradability, and biocompatibility. Therefore, SF has attracted interest in the development of sustainable biodevices, especially for emergent bio-electronic technologies. To expand the function of current silk devices, the SF characteristic sequence has been used to synthesize recombinant silk proteins that benefit from SF and other functional peptides, such as stimuli-responsive elastin peptides. In addition to genetic engineering methods, innovated chemistry modification approaches and improved material processing techniques have also been developed for fabricating advanced silk materials with tailored chemical features and nanostructures. Herein, this review summarizes various methods to synthesize functional silk-based materials from different perspectives. This review also highlights the recent advances in the applications of natural and recombinant silks in tissue regeneration, soft robotics, and biosensors, using *B. mori* SF and silk-elastin-like proteins (SELPs) as examples.

## Introduction

The increasing demands for advanced healthcare have driven the development of numerous functional biodevices to potentially address multiple clinical-related challenges and establish personalized healthcare (Zhao et al., 2020). These biodevices, including biosensors (Tao et al., 2012a; Yang N. et al., 2019), implantable devices (Li et al., 2021), electronic skins (Yang J. C. et al., 2019), and microfluidic devices (Dong et al., 2019), have been applied in realms such as precision diagnostics, health monitoring, drug delivery, and tissue engineering. Traditional implantable and wearable biodevices are made of rigid materials that lack compliance with targeted body tissues. The mismatches in the dimensions, interfaces, and motions may lead to foreign body reactions and limited performances (Cole et al., 2021). Hence, a trend towards developing soft and deformable biodevices has become predominant.

Nature provides enormous sources of materials and design concepts that are beneficial for developing novel biodevices (Pradhan et al., 2020). Biobased materials obtained from animals and plants, such as proteins and polysaccharides, are promising building blocks for “green” biodevices. Natural fibrous proteins usually consist of linear peptide chains with specific sequences of amino acids. These proteins exhibit distinct chemistries and can form ordered structures under certain conditions. Because of the dynamic non-covalent bonds among amino acids, fibrous proteins can self-assemble to form supramolecular structures which may eventually lead to hierarchical structure and interconnected networks. These unique properties of natural fibrous proteins enable the possibility of reprogramming material formats via adjusting protein sequences. Engineering these biobased materials for tailored chemical features and biomimetic nanostructures offers new paths towards next-generation functional devices.

Recently, silk has come to the forefront of sustainability research due to its low global carbon footprint (Tao et al., 2012b; Irimia-Vladu, 2014). Silks are fibrous proteins that are spun into fibers by silkworms and spiders. Silk is one of the most studied natural fibrous proteins, which provides a peptide template for advanced material design. The main motivations for using silk-derived materials are their biocompatibility, programmable biodegradability, large-scale production, and versatility. By altering amino acids using synthetic biology techniques, silk can be used as building blocks to fabricate novel biodevices with sophisticated nanostructures. From a materials science perspective, silks possess extraordinary physical properties, including remarkable strength, toughness, optical transparency, tailorable biodegradability, and biocompatibility (Guo et al., 2020b). The toughness of *B. mori* silk (70–87 MJ/m^3^) even exceeds Kevlar (50 MJ/m^3^) (Guo et al., 2020b). Historically, silk has been used by the textile industry. With an improved understanding of the relationship between silk amino acid sequences, protein structure, processing method, and material function (Huang et al., 2017a), “ancient” silk materials have been transformed into new material formats, including nanoparticles, thin films, hydrogels, and 3D micro-structured scaffolds, for a range of biodevice applications (Huang et al., 2018). In the past few decades, silk-based biodevices were limited to 2D planar shapes with the design concept of “bioresorbable electronics,” among which silk-based layers acted as inert supporting materials because of their outstanding mechanical strength of silk films (Wang et al., 2011). With the emergence of various novel fabrication technologies, such as lithography and 3D printing, silk-based biodevices in recent decades have evolved to be more conformable and possess precise nanopatterns with additional properties such as being self-healable and stretchable (Tao et al., 2012b; Wang C. et al., 2019; Yang J. C. et al., 2019). These biodevices have been considered candidates for biosensors, wearable devices, and transistors. Moreover, with the demand for enhancing device compliance, 2D silk-based biodevices have been reshaped with 3D geometries to match the dimensions and curvatures between tissues and biodevices. The nanoscale control of 3D silk biodevices also endows the materials with biomimetic nanostructures suitable for cell growth (Guo et al., 2017). Therefore, in recent years, enhanced 3D silk biodevices have been adapted in different areas for regenerative medicine, such as skin repair (Lu et al., 2018), neovessel remodeling (Wang Z. et al., 2020), and microfluidic cell culture model (Zhao et al., 2016). Nowadays, the next generation silk biodevices are expected to be self-adaptive that can sense and respond to the stimulus from the microenvironment. These “smart” biodevices can perform designed reconfiguration under stimuli, appears as morphological changes or resistance changes, thus being used as cell culture devices (Parker et al., 2020), sensors (Wang Q. et al., 2019), and actuators (Wang Y. et al., 2020). In addition, translational research on silks also led to various FDA-approved biomedical devices, such as silk sutures (Surusil^®^, Suru; Sofsilk™, Covidien) and soft tissue scaffold (Seri^®^ Surgical Scaffold, Allergan). With the advent of advanced genetic engineering techniques, silk-based materials have been designed *de novo* to achieve new functions with the aid of computer simulations (Huang et al., 2017a). In particular, new recombinant silks were built from the molecular level to possess both good mechanical properties from silk domains and target functions from other functional peptides, such as stimuli-responsive properties from elastin peptides (Huang et al., 2016). Inspired by the hydration-driven botanic systems, stimuli-responsive recombinant silk-elastin-like proteins (SELPs) were designed *de novo* and combined with cellulose nanofibers (CNFs) to fabricate biomimetic actuators (Wang Y. et al., 2020). Programmable and reversible deformations in response to external stimuli were achieved by the SELP/CNF actuator towards applications in the fields of *in vivo* biomedical soft robotics. In summary, natural silk and recombinant silk materials have established a promising material platform for the next generation of biodevices.

**FIGURE 1:**
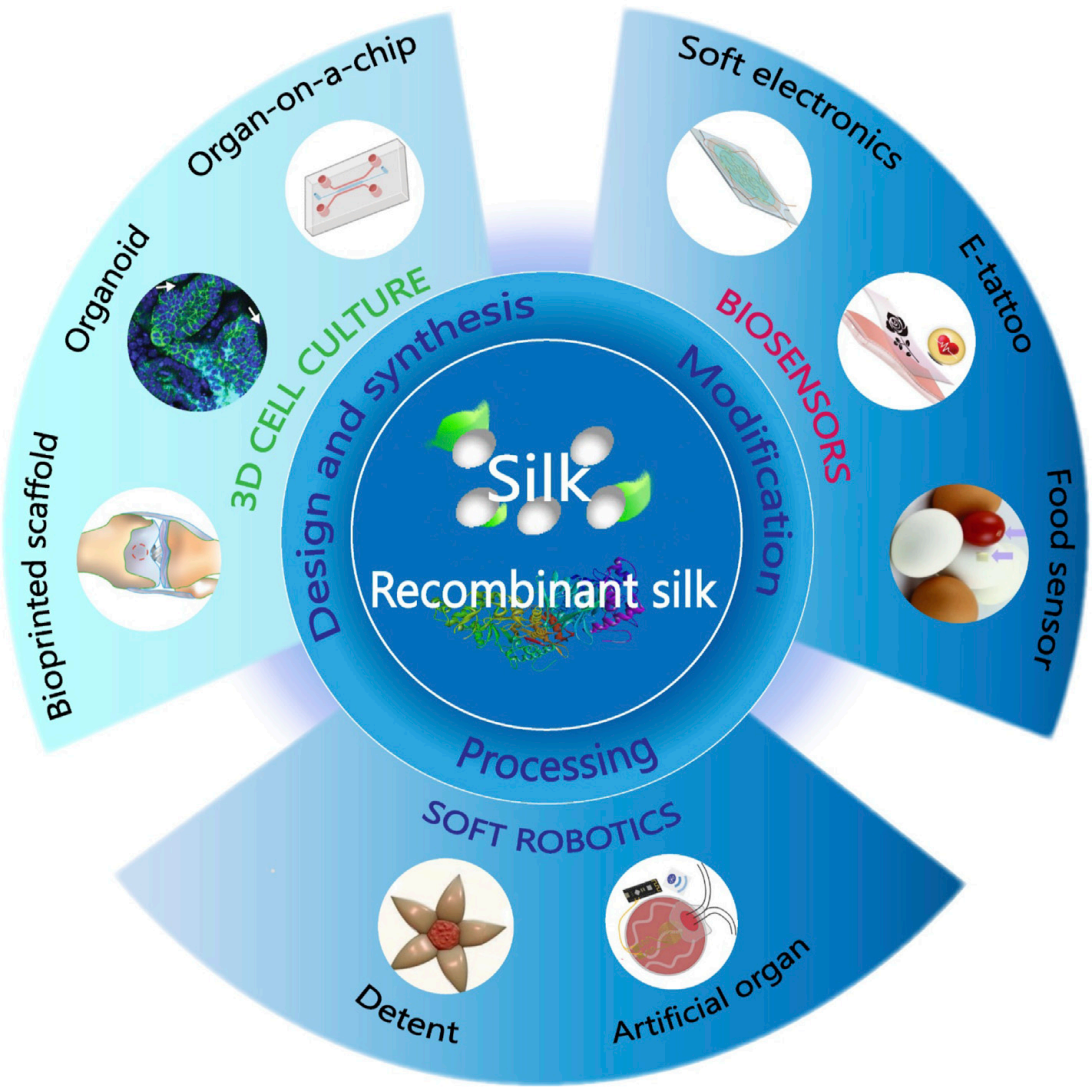
Biodevices fabricated from natural and recombinant silk. Using innovative chemical modifications and improved material processing techniques, the synthesized materials will be functionalized and fabricated into different forms and patterns for biodevice applications, including 3D cell culture, soft robotics, and biosensors. Adapted with permission from ref (Tao et al., 2012a; Yang et al., 2018; Wang Q. et al., 2019; Gupta et al., 2019; Wang Y. et al., 2020; Hayward et al., 2021; Yang et al., 2021). Copyright 2012 WILEY-VCH, 2018 WILEY-VCH, 2019 WILEY-VCH, 2020 National Academy of Sciences, 2021 American Chemical Society.

This review mainly focuses on sustainable biodevices fabricated using silk-based materials, including natural silk and recombinant silk (Figure 1). In the first part of the review, the material synthesis method of natural silk and *de novo* design of recombinant silk are summarized. Following this section, innovative chemical modifications and improved material processing techniques for fabricating advanced silk materials with tailored chemistry and biomimetic nanofeatures are investigated. Finally, advances in three application fields of silk-based biodevices are reviewed, including tissue regeneration, soft robotics, and biosensors.

## Silk-Based Materials

### Materials Design and Synthesis

**FIGURE 2:**
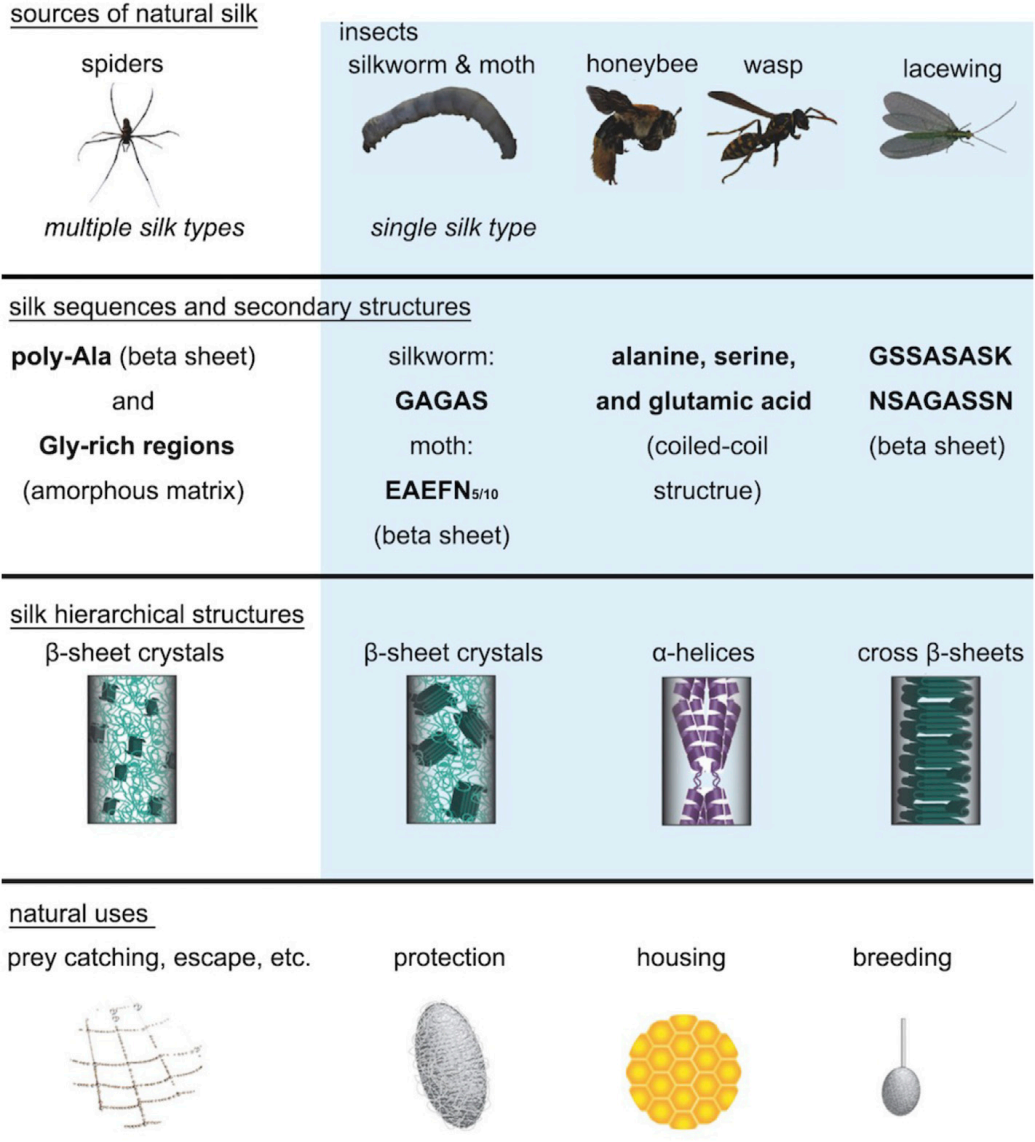
Natural silks derived from different animal species. The silk structures of spiders, silkworms, moths, honeybees, wasps, and lacewings show significant differences between each other. Spiders, silkworms, and moths all have silks in which β-sheet crystals are embedded in the amorphous matrix. However, the β-sheet crystals in spider silk are much smaller than the β-sheet crystals in silkworm silk and are highly aligned along the fiber axis. Honeybee silk and wasp silk mainly consist of α-helix structures, while lacewing silk consists of cross β-sheet structures. These silk secondary structures are highly related to their properties and functions. Adapted with permission from ref (Aigner et al., 2018). Copyright 2018 WILEY-VCH.

Different species of insects and spiders produce a large variety of silks in nature. Silkworms construct cocoons out of silk fibers as a protective strategy against changes in the natural environment or against predators during the vulnerable stage of pupation. Spiders spin various types of silk for constructing webs, wrapping of prey, and as a lifeline. Most natural silks are composed of structural proteins which are rich in alanine, serine, and/or glycine, and thus natural silks have either a high content of β-sheets or α-helices (Aigner et al., 2018). Due to the difference in primary sequences, secondary structures, and hierarchical structures, natural silks show significant differences in materials properties and functions (Figure 2). In general, spider and silkworm silks have extraordinary mechanical stiffness due to the nanoscale confinement of aligned β-sheet nanocrystals in the semi-amorphous matrices; lacewing silk has high bending stiffness due to the cross β-sheet structures; and honeybee silk exhibits distinct linear and brittle elastic mechanical behavior due to the coiled-coil structures by α-helices (Aigner et al., 2018). This review primarily focuses on research related to silkworm silk from the silk moth *B. mori* because of its good material properties, easy availability in nature, and potential for translational applications.

**FIGURE 3:**
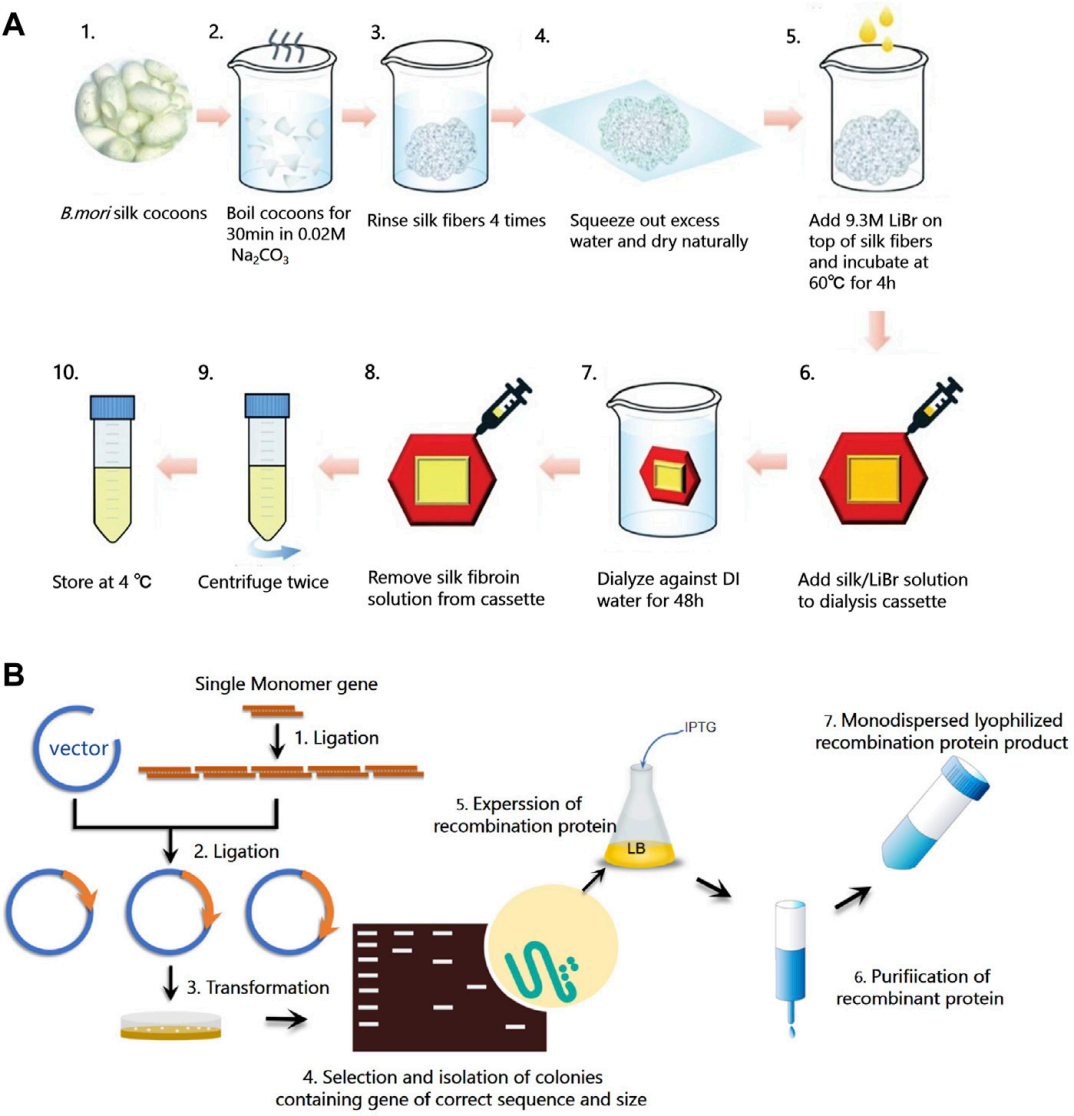
Synthesis methods of silks. **(A)** Schematic diagram of silk fibroin extraction procedure. Silk fibroin (SF) can be extracted from native *B. mori* silk following a three-step process: fiber degumming (step A1-4), fiber dissolution (step A5), and solution purification (step A6-10). Degummed silk fiber and regenerated silk fibroin solution can be obtained from this procedure. Adapted with permission from ref (Malinowski et al., 2019). Copyright 2019 Springer Nature. **(B)** Schematic diagram of the synthesis procedure of recombinant silks, including plasmid construction (step B1-2), host transformation (step B3-4), protein expression (step B5), and purification (step B6-7).

Native *B. mori* silk is composed of two types of proteins, silk fibroin (SF) and sericin, in an approximate mass ratio of 7:3. Sericin is the glue-like protein that serves to maintain the shape of the cocoon, while SF is a semicrystalline biopolymer with maximum crystallinity of about 55% (Huang et al., 2018). SF can be extracted from native *B. mori* silk following a three-step process (Rockwood et al., 2011; Malinowski et al., 2019): fiber degumming, fiber dissolution, and solution purification (Figure 3A). In the degumming process, sericin are removed from silk fibers by boiling the cocoons in a basic solution. The peak molecule weight of SF protein decreases with the increasing degumming time (Partlow et al., 2016). In addition to degumming time, the alkali agents used in the degumming process and temperature also affect the degradation and thus molecular weight distribution of SF, resulting in changes of material properties such as mechanical strength, optical property, and nanostructure (Partlow et al., 2016). In the dissolution process, hydrogen-bonded crystalline structures in silk proteins are disrupted using denaturing agents. Commonly used denaturing agents of SF include lithium bromide, calcium chloride, formic acid, and hexafluoro-2-propanol (Guo et al., 2020b), which can convert β-sheet structures into α-helices and random coils (Huang et al., 2017b). In the purification process, aqueous SF solutions are purified by dialysis against water to remove the denaturing agents. Then, a variety of aqueous processing methods can be used to fabricate SF solution into a broad range of material formats, including hydrogels, sponges, nano- or microparticles, fibers, films, tubes, microspheres, and powder (Rockwood et al., 2011; Qi et al., 2017), with potential for realizing various silk-based biodevices.

Common problems of many natural materials, especially functional proteins, are their impurities, the risk of immune responses, and the difficulties in mass-production (Aigner et al., 2018). To find an alternative routes to synthesize protein materials and expand the function of current silk proteins for sustainable biodevices, genetic engineering methods have been used to synthesize recombinant silks that consist of silk characteristic sequences and other functional peptides (Figure 3B). To create dynamic and environmentally responsive materials with good mechanical properties, SELPs with different numbers of silk motifs (GAGAGS) and various elastin motifs (G ***X*** GVP) were synthesized by a high throughput method (Huang et al., 2017a). The silk motifs contribute to the formation of antiparallel β-sheets, which correlates with high tensile strength, while the elastin motif contributes to the stimuli-responsive properties (Huang et al., 2017a). By tailoring the ratio between silk to elastin domains, adjusting the SELPs polymer length, or changing the characteristic “X” amino acid in the elastin domains, SELPs can be designed *de novo* to be endowed with various stimuli-responsive properties and mechanical strengths (Huang et al., 2017a). Moreover, by introducing cell recognition motifs, such as arginine-glycine-aspartic acid (RGD) or isoleucine-lysine-valine-alanine-valine (IKVAV), cell adhesion can be facilitated on recombinant silk materials (Debabov and Bogush, 2020). Furthermore, akin to the cell adhesion sequence, by mutating certain amino acid residues or encoding functional peptides to the silk domain, new functions can be endowed in recombinant silks. For example, tyrosine crosslinking sites were encoded in SELP for the fabrication of smart hydrogels, which can respond to environmental stimuli, including changes in temperature, pH, and biological signals (Huang et al., 2016). C-terminal adenosylcobalamin binding domain (CarH_C_) was genetically encoded in SELP sequence to obtain photoresponsive SELP hydrogels (Narayan et al., 2021). In summary, using genetic engineering, new properties and enhanced functions, such as stimuli-responsive properties, cell adhesion, antibacterial property, enzymatic activity, fluorescence, and biomineralization, can be encoded into recombinant silks (Debabov and Bogush, 2020; Lee et al., 2020). Recombinant silks have been shown to be generally versatile in terms of tunable material properties and tailorable functions.

**FIGURE 4:**
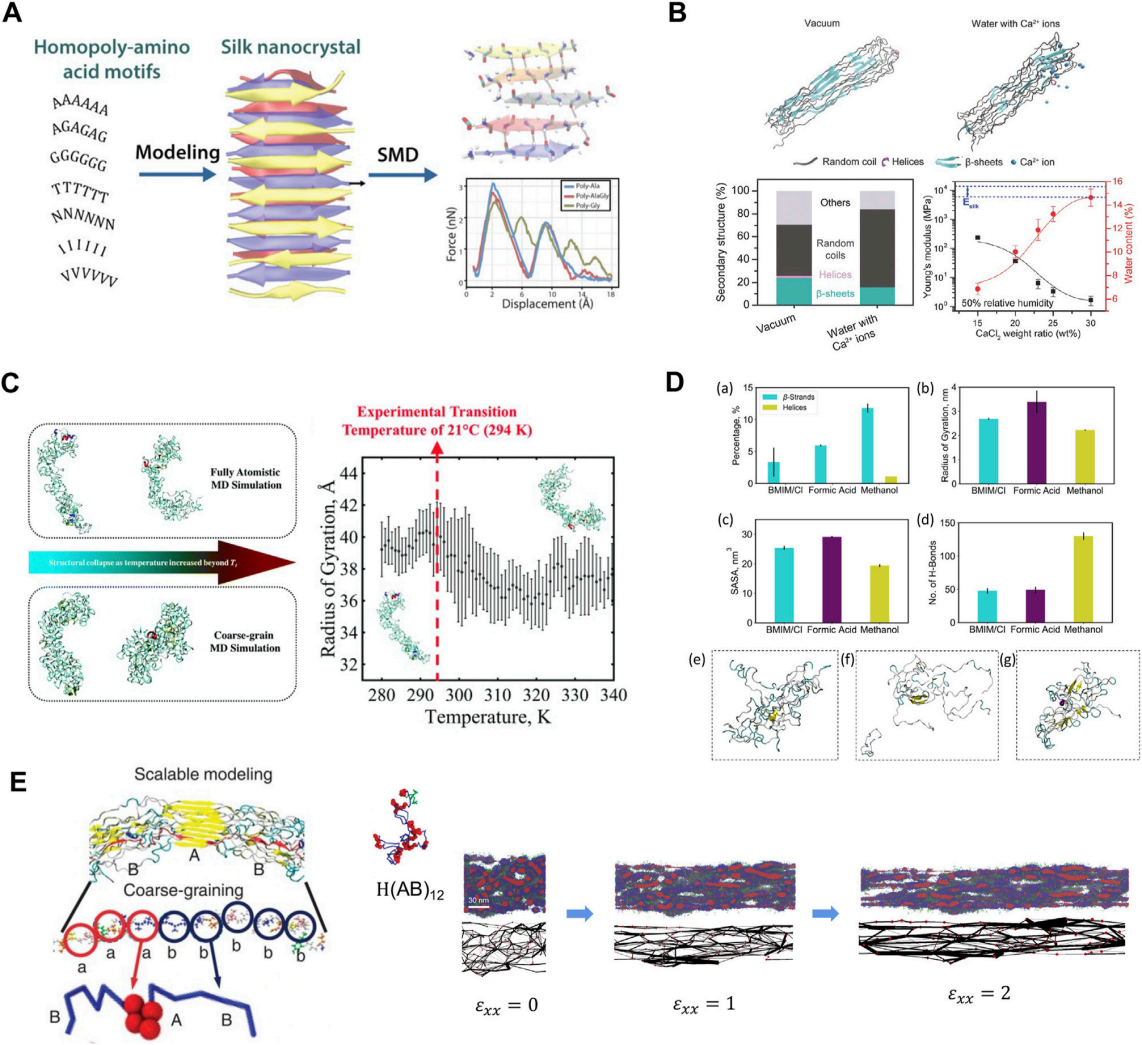
Multiscale modeling facilitates the design of new silks. **(A)** Models of β-sheet nanocrystals with various representative small/polar/hydrophobic amino acid repeats. Simulation results showed that homopolymers of alanine and alanine–glycine sequence motifs have better nanomechanical properties than other modeled structures. Adapted with permission from ref (Verma et al., 2021). Copyright 2021 American Chemical Society. **(B)** Representative snapshots of silk protein in vacuum and water with Ca^2+^ ions. The secondary structures showed water and Ca^2+^ ions introduce extensible structures in silk to reduce Young’s modulus and improve stretchability. Adapted with permission from ref (Chen et al., 2018). Copyright 2018 WILEY-VCH. **(C)** Structural conformations of SELP captured by REMD and CGMD. A distinct structural transition temperature was captured in terms of the radius of gyration. Adapted with permission from ref (Yeo et al., 2018). Copyright 2018 Royal Society of Chemistry. **(D)** REMD simulations obtained SF structure with the influence of BSEISA. Adapted with permission from ref (Shu et al., 2021). Copyright 2021 American Chemical Society. **(E)** Schematic of silk CG model and the silk network deformation process under various tensile strains. Adapted with permission from ref (Lin et al., 2015). Copyright 2015 Springer Nature.

Simulation-aided recombinant silk design, accompanied by continuing developments in computing hardware, software, and algorithms, can help to accelerate silk product development further. A prime example is the use of molecular dynamics (MD) simulations to determine the deformation behaviors of silk β-sheets. Such studies guided the modulation of stiffness/softness in silk-based materials from molecular perspectives (Zhai et al., 2020), often as a design goal to exploit silk properties. Early studies applying MD simulations revealed that the exceptional stiffness in multilayered silk β-sheets was due to the “self-healing” ability when hydrogen bonds sequentially break and reform (Keten et al., 2010). Subsequent computational studies targeted this mechanism to optimize material designs for various applications. For example, altering silk β-sheet sequences with alanine residues resulted in higher mechanical strength and toughness, whereas sequences with high poly-polar/hydrophobic residues formed β-sheets with lower mechanical tensile strengths (Verma et al., 2021) (Figure 4A). Water molecules and Ca^2+^ ions also compete for hydrogen bonds between silk β-sheets to weaken or amorphize the silk structures (Chen et al., 2018; Haskew et al., 2021) (Figure 4B). These findings can inspire the design of silk-based materials with tunable mechanical properties. Other than silk β-sheet blocks, MD simulations are frequently coupled with experimental characterization to validate outcomes and provide feedback for further optimization. Common features captured by MD simulations in recombinant silk-based materials include, but are not limited to, structural conformations and mechanical properties. Through advanced sampling methods, such as replica exchange molecular dynamics (REMD) (Sugita and Okamoto, 1999), the folded conformations of globular and fibrous proteins can be obtained rapidly and accurately in various environmental conditions and solvents. These protein structures can then be analyzed further to determine properties such as binding affinity and structural transformations. Relevant examples include SELP (Huang et al., 2015; Huang et al., 2016; Yeo et al., 2018) **(** Figure 4C **)** and binary solvent-exchange-induced self-assembly (BSEISA)-SF (Shu et al., 2021) (Figure 4D). Furthermore, coarse-grained (CG) MD simulations with mature CG schemes for silk protein can capture the formation of micelles and polymer networks on the mesoscale (Lin et al., 2015). Thus, the collective mechanical properties for clusters of SF can be obtained by directly deforming the simulation box to introduce a stain (Rim et al., 2017; Patel et al., 2020), where the elastic modulus and tensile strength can be qualitatively solved to validate and explain experimental results (Figure 4E).

### Materials Modifications

**FIGURE 5:**
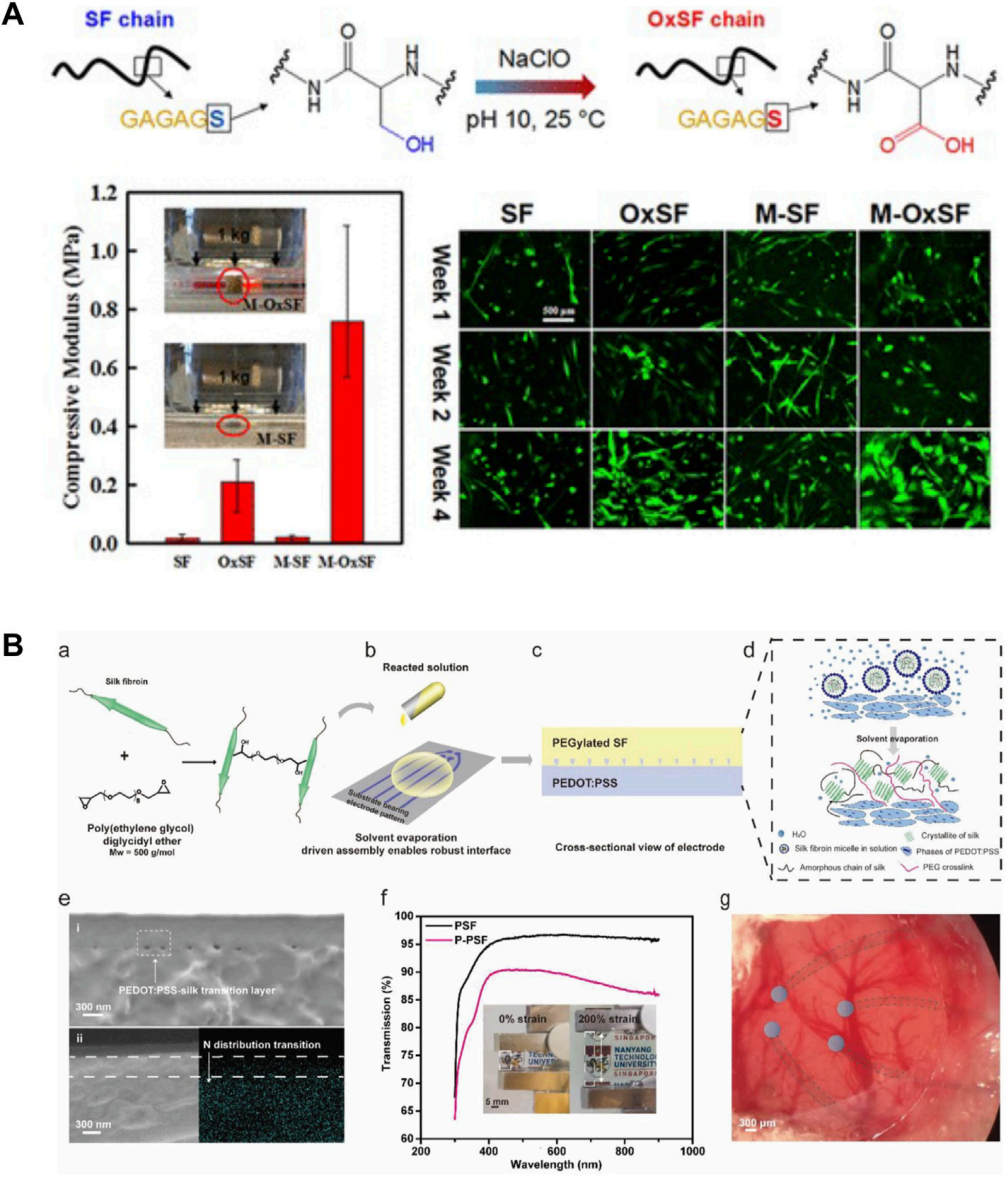
Material modification strategies on silk-based materials. **(A)** Silk fibroin (SF) was oxidized using sodium hypochlorite (NaClO). The oxidized silk (OxSF) scaffolds showed 10 times higher compressive modulus (211 ± 75 kPa) in the hydrated state than the SF scaffolds. Meanwhile, the mineralized OxSF scaffolds (M-OxSF) possessed a greater modulus of 758 ± 189 kPa. Human bone marrow-derived mesenchymal stem cells (hMSCs) proliferated and differentiated on the oxidized scaffolds *in vitro*. Adapted with permission from ref (Zheng et al., 2016). Copyright 2016 American Chemical Society. **(B)** Design of transparent, stretchable, and implantable SF-based hydrogel electrode. SF was crosslinked with poly (ethylene glycol) diglycidyl ether and then was subsequently poured onto PEDOT:PSS to form a thin film silk electrode. Solvent evaporation allowed more interactions between silk and PSS. The resultant electrodes were highly transparent and stretchable. Adapted with permission from ref (Cui et al., 2021). Copyright 2021 WILEY-VCH.

Silk proteins are primarily composed of non-reactive amino acids, such as glycine and alanine, and a relatively small quantity of reactive amino acids, such as serine, threonine, aspartic, glutamic acid, and tyrosine. The amino acid composition of SF includes 45.9% glycine, 30.3% alanine, 12.1% serine, 5.3% tyrosine, 1.8% valine, and 4.7% of the other 15 amino acid types (Huang et al., 2018). The presence of reactive amino acids in silks allows chemical modification strategies to be utilized to tailor silk material properties. Tyrosine (Murphy et al., 2008) and serine residues (Zheng et al., 2016) are the most abundant reactive sites in silks, which can increase the amount of functional group incorporation and the homogeneity of modification distribution along the protein chain. Common chemical modification strategies on tyrosine include diazonium coupling, cyanuric chloride-activated coupling, oxidation, and sulfation (Vepari and Kaplan, 2006; Murphy and Kaplan, 2009), while common chemical modification strategies on serine include oxidation and sulfation (Murphy and Kaplan, 2009; Zheng et al., 2016). These reactions on silks provide a simple route for controlling protein structure, property, and function. For example, diazonium coupling was used to functionalize tyrosine residues and add spectral-color-responsive pH sensitivity in SF for optofluidic devices (Tsioris et al., 2010). In another study, SF films were also successfully modified with various non-natural functional groups, such as sulfonic acid and alkyl functional groups, by diazonium coupling (Murphy et al., 2008). This study suggested that the surface chemistry, especially the overall hydrophilicity, can be tuned to control the growth rate and morphologies of human bone marrow-derived mesenchymal stem cells. Additionally, serine residues in SF were oxidized with sodium hypochlorite to promote the formation of β-sheet structures and *in vitro* mineralization in SF (Zheng et al., 2016). This study suggested that the compressive modulus of oxidized SF scaffold is 10 times higher compared with the un-modified SF scaffold due to the increased β-sheet content. The oxidized SF scaffold also supported the proliferation and differentiation of human bone marrow-derived mesenchymal stem cells *in vitro* (Figure 5A) (Zheng et al., 2016). Chemical modifications have been shown to change the chemistry of amino acid side chains and therefore change the protein chain folding and alter important material features, such as hydrophobicity, surface charge, β-sheet content, and crosslinking density (Murphy and Kaplan, 2009).

In addition to these amino acid modifications, the hybridization of silk with other organic and inorganic components provides alternative strategies to functionalize silks with enhanced properties. Crosslinking, grafting, blending, and genetic engineering methods have been widely applied to synthesize hybrid silk materials, such as protein-polymer composite, protein-polymer conjugates, protein nanocomposite, and recombinant proteins. For example, SF was crosslinked with poly (ethylene glycol) diglycidyl ether to reduce the β-sheet content in SF films (Cui et al., 2021). This modification improved the stretchability of silk to >400% under wet conditions, thus enabling the fabrication of a highly stretchable and transparent neural electrode (Figure 5B). The platelet adhesion of silk was significantly decreased by grafting with 2-methacryloyloxyethyl phosphorylcholine (Furuzono et al., 2000). This modification eliminates the inflammatory reactions caused by platelet, monocyte, and macrophages, thus improving the material blood compatibility. SF has also been functionalized with conductive nanomaterials to improve the electrically conductive properties for the fabrication of biodevices. Graphene was incorporated into silk inks and formed electrically conductive paths to fabricate graphene/silk/Ca^2+^ E-tattoo. The printed E-tattoo was self-healable and highly flexible, thereby allowing it to be conformally attached to human skin for detecting relative resistance changes as biosensors (Wang Q. et al., 2019). Overall, modification of silks with polymers and nanoparticles has been shown to expand material characteristics and enhance the electrical, mechanical, thermal, and biological performance of silk biomaterials.

### Materials Processing

**FIGURE 6:**
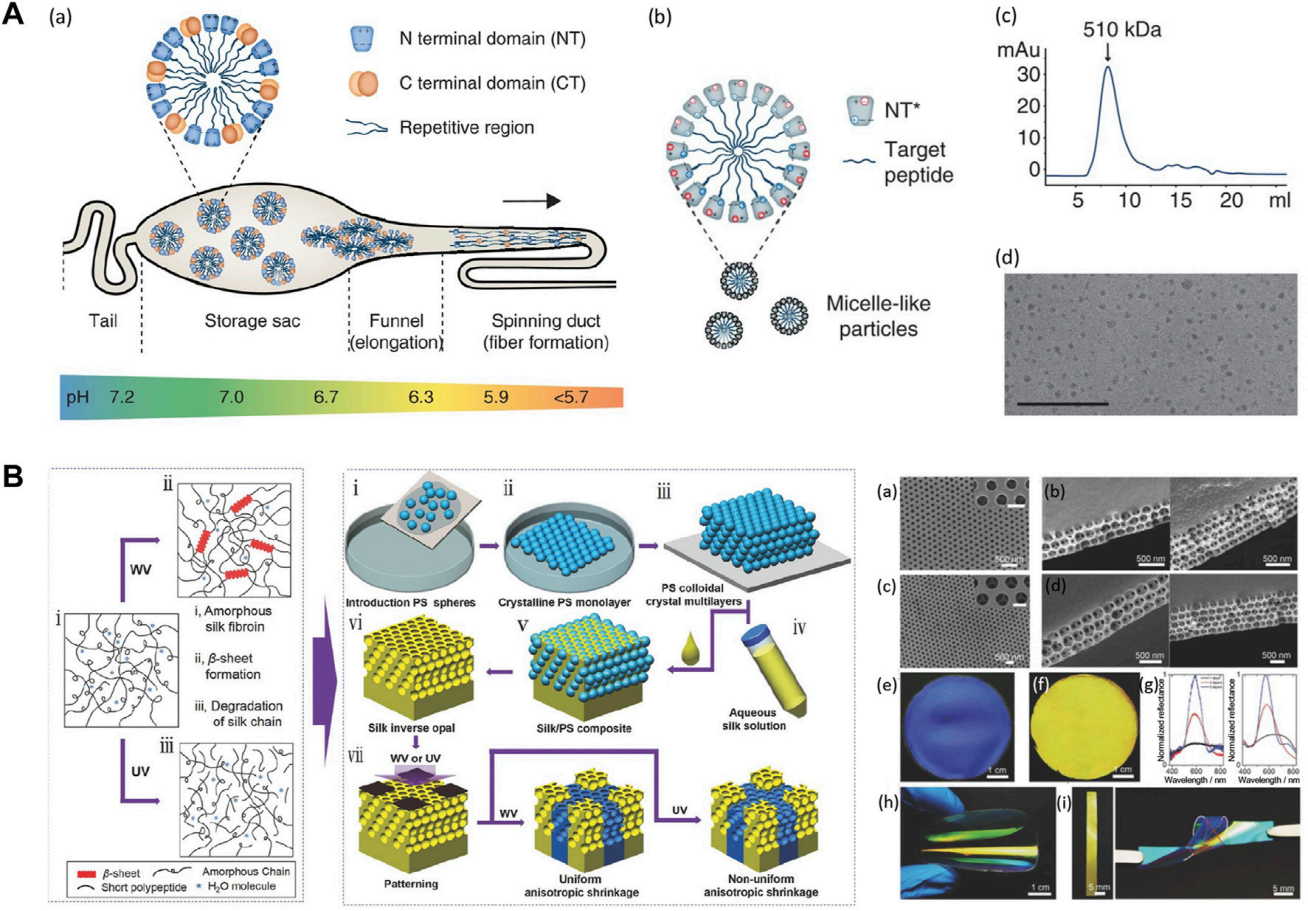
Material processing strategies on silk-based materials. **(A)** Design of the spider silk spinning systems. **(A)** Spidroins are synthesized in the tail region and stored in the storage sac where pH = 7.0. Proteins form micelles to prevent premature aggregation. The hydrophilic NT domains have dipolar charge distribution, represented by + and - symbols. Spidroins are self-assembled when going through the spinning duct due to the pH changes and shear forces. **(B)** NT can be used as a fusion tag to mediate protein solubility and protect hydrophobic regions from aqueous solutions. The spidroins with mutant NT* can adjust solubility over a wider pH range based on the reduced dipolar charge distribution of NT* than NTwt. **(C)** Size exclusion chromatography shows purified amphiphilic fusion protein form 510 kDa assemblies. **(D)** TEM image shows that spidroins micelle-like particles are around 10–15 nm in size. Adapted with permission from ref (Kronqvist et al., 2017). Copyright 2017 Springer Nature. **(B)** Fabrication of silk inverse opals. Using colloidal crystals composed of PS spheres as SIOs templates, SF solution was infiltrated into PS SIOs templates and allowed to dry. The resultant silk/PS composite film was immersed into toluene to dissolve the PS substrate. Patterned silk inverse opals are formed after treating with water vapor (WV) and UV light. The diameters of PS templates were 210 nm and 300 nm. Adapted with permission from ref (Wang et al., 2017). Copyright 2017 WILEY-VCH.

High resolution and high throughput micro-nano fabrication technology are playing an increasingly important role in the development of sustainable biodevices. In nature, silkworms and spiders have developed sophisticated spinning systems to fabricate silk fibers via a protein self-assembly process from highly concentrated protein solutions (Figure 6A) (Kronqvist et al., 2017). Inspired by the natural biological spinning process, a variety of solution assembly techniques have been developed to precisely control the hierarchical structure and pattern the topology of silk biomaterials into a range of new material formats, such as 0D nanoparticles, 1D nanofibers, 2D thin films, and conformal coatings, or 3D porous scaffolds and hydrogels (Huang et al., 2018). The most popular wet-chemistry approaches to process silks include self-assembly, bio-inspired spinning, casting, spin coating, layer-by-layer assembly, soft lithography, vacuum-assisted filtration, 3D printing, and gelation (Tao et al., 2012b; Guo et al., 2020b). The resolution of these fabrication approaches is highly dependent on the physicochemical properties of the silk solutions, such as intra- and intermolecular interactions between silk motifs, concentration, and viscosity. Silk nanoparticles are an interesting form that can be fabricated using self-assembly strategies. Inspired by the alternately arranged hydrophilic and hydrophobic chain segments in the silk molecules, recombinant silks were designed into amphiphilic molecules with the ability to self-assemble into nanoparticles, micelles, or fibers with hierarchical structures. Amphiphilic SELPs were self-assembled into micellar-like nanoparticles with mucoadhesive properties. The results indicated that SELP-based nanoparticles could increase the retention rate of the particles in mucosal environments, showing great potential for transmucosal drug delivery (Parker et al., 2019). Silk films are typically prepared by casting silk solution into a pre-designed mold with subsequent controlled solvent evaporation. The topographies on the pre-designed mold can be replicated in the optical transparent silk films down to the feature sizes less than 10 nm (Tao et al., 2012b). Soft lithography was applied to fabricate silk inverse opals (SIOs) by infiltrating silk solution into a pre-templated 3D poly (methyl methacrylate) (PMMA)/polystyrene (PS) sphere array (Figure 6B) (Wang et al., 2017). The SIOs film can optically detect and record the surrounding humidity for applications in optics, electronics, and sensors (Wang et al., 2017). Hydrogels are an important 3D material format that can be fabricated by wet-chemistry approaches, including enzyme-catalyzed crosslinking, physical crosslinking, photo-crosslinking (Kundu et al., 2012; Cui et al., 2020), electro-gelation, vortex, as well as sonication (Rockwood et al., 2011; Partlow et al., 2014). The speed of the gelation process can be optimized by adjusting reaction conditions, including temperature, pH, ion concentration, shear force, protein molecular weight, and solution concentration (Kim et al., 2016; Qi et al., 2017). 3D silk hydrogel microfluidic devices were constructed using enzyme-catalyzed crosslinking and layer-by-layer assembly method (Zhao et al., 2016). This aqueous-based processing technique enabled the incorporation of active biological substances in silk hydrogel microfluidic chips for active diagnostic devices and on-chip cell sensing systems.

Recently, thermal processing methods (Amsden et al., 2010; Guo et al., 2020a) have been developed as a rapid, low cost, and high throughput strategy for the fabrication of silk materials. During thermal processing, the temperature of silks was rapidly raised above the water-associated glass transition temperature (Tg), causing a transition from brittle, glassy state to flexible rubbery state in the amorphous region in silks, and thus allowing reflow of protein chains on the nanoscale for material fabrication. Nanoimprint lithography was applied to transfer sub-100 nm features onto silk films for biophotonic sensing applications (Amsden et al., 2010). Thermoplastic molding methods was also developed recently to shape amorphous silk nanomaterials into medical devices such as bone screws and ear tubes (Guo et al., 2020a). These thermal processing methods can be tuned to incorporate bioactive molecules by adjusting the absorbed moisture content in silks. Comparing with solution assembly techniques, thermal processing can easily reach a high protein density for the fabrication of tough biomaterials. Thermal processing techniques also overcome the current limitations associated with solution-based processing approaches, such as silk protein solubility and protein stability in solution.

## Applications

### 3D Cell Culture

Tissue or organ dysfunction remains a major health problem of the aging and diseased population. Among various solutions, tissue engineering provides a powerful tool to repair, replace or regenerate damaged tissues and organs. Cell culture systems are one of the major *in vitro* tools used in tissue engineering. 2D cell culture systems have been used as traditional cell culture technique due to its convenience, ease of operation, high cell viability, and low cost (Lee et al., 2008). However, existing 2D cell culture models poorly mimic the conditions *in vivo*, thus having many limitations in understanding cell-cell interactions, cell-extracellular matrix (ECM) interactions, and cell polarity. Recent studies have suggested that three-dimensional (3D) cell culture systems, in contrast to the 2D culture system, represent more accurately the actual microenvironment of ECM, and thus the behavior of 3D-cultured cells is more reflective of *in vivo* cellular responses (Han et al., 2020). Therefore, understanding and creating biologically compatible 3D matrices is crucial to making progress toward the design of new cell culture systems.

**FIGURE 7:**
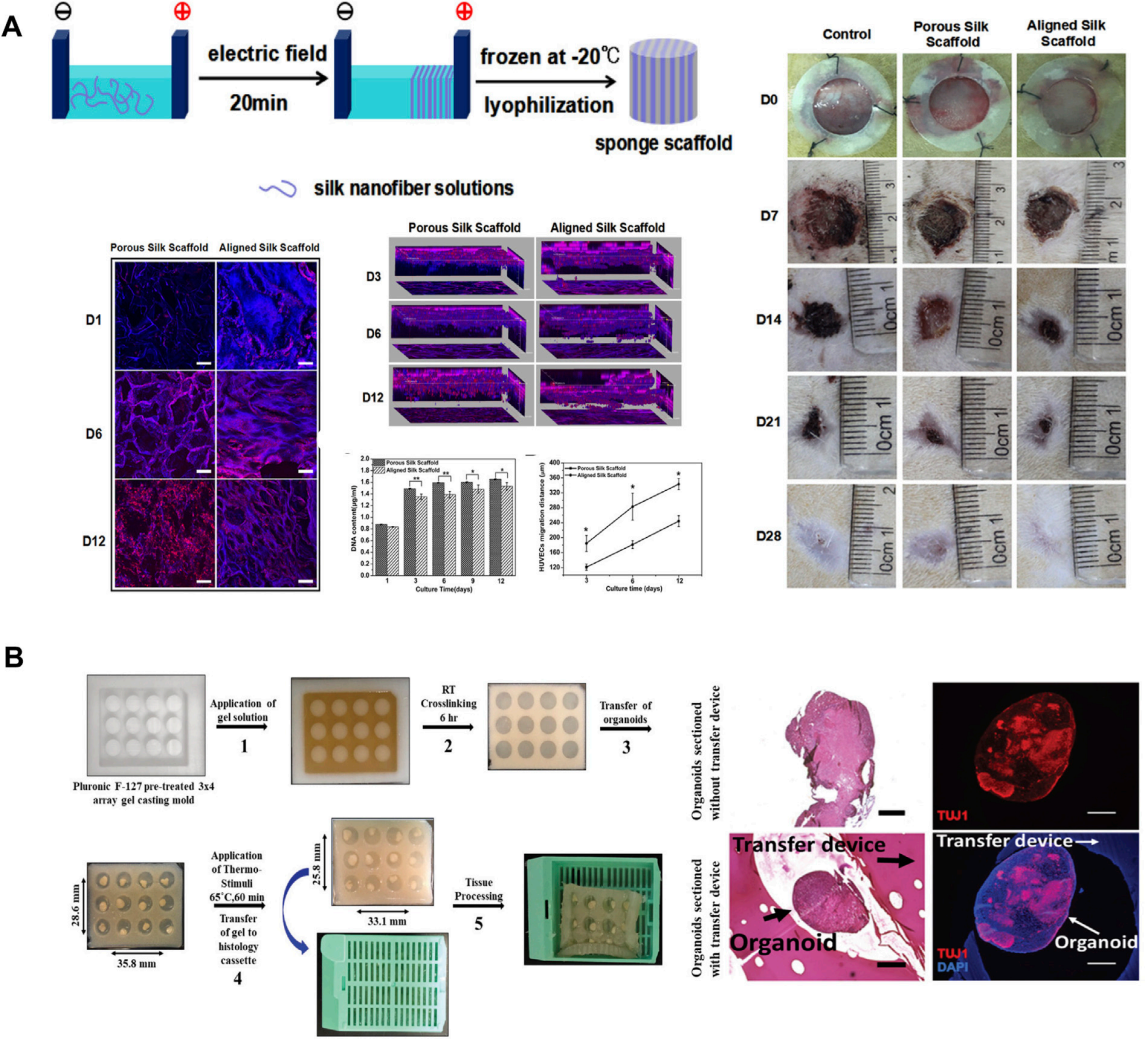
Silk-based biomaterials in 3D cell culture. **(A)** The silk nanofibers were aligned in an electric field and then lyophilized to obtain aligned silk nanofiber scaffolds with biomimetic nanostructures. Human umbilical vein endothelial cells migrated faster and deeper on the aligned silk scaffold because of the aligned hierarchical structures. Rat skin full-thickness defect model suggested that aligned silk scaffolds provided better microenvironments for wound closure than porous silk scaffolds. Adapted with permission from ref (Lu et al., 2018). Copyright 2018 American Chemical Society. **(B)** Schematic diagram of thermo-responsive SELP devices for high throughput histology analysis. Histological analyses and immunofluorescent staining of cerebral organoids suggest that the SELP embedded organoids appear to maintain more defined edges, present fewer tears, and show more compact tissue organization. Adapted with permission from ref (Parker et al., 2020). Copyright 2020 WILEY-VCH.

Silk-based materials are ideal candidates for generating 3D cell culture systems due to their biocompatibility, excellent mechanical properties, controllable degradation rate, and the ability to be functionalized and processed into multiple material formats under aqueous conditions (Rockwood et al., 2011). Recently, nanoscale control of 3D silk materials has shown great promise to regulate the matrix features, including the porous structure and aligned nanostructure, which are important in regulating cell and tissue outcomes for tissue regeneration. For example, two-layer small-diameter vascular grafts were designed and fabricated using a two-step cross-electrospinning method for neovessel remodeling (Wang Z. et al., 2020). The inner layer consisted of heparinized silk fibroin and polycaprolactone with oriented nanostructure, while the outer layer consisted of PCL with a vertically porous structure. The oriented structure and anticoagulant bioactivity presented synergistic effects on rapid vessel endothelialization and inhibition of platelet adherence. Anisotropic porous SF scaffolds were fabricated by aligning SF nanofibers in an electric field followed by lyophilization (Lu et al., 2018). The anisotropic features in silk nanofiber scaffolds with hierarchical ECM-like morphology effectively promoted cell migration and the healing of full-thickness skin wounds (Figure 7A) (Lu et al., 2018). Perfusable alginate/silk fibroin (Alg/SF) scaffolds with complex microfluidic channels were fabricated using the coaxial extrusion-based bioprinting method (Li et al., 2020a). These interconnected microchannels play an essential role in elevating cell viability and facilitating the vascularization process (Li et al., 2020a). Similarly, silk fibroin–chitosan (SF-CS) scaffolds were designed and fabricated with predefined microfluidic channels and porous structures using the ice-template-induced (ITI) method. The complex microfluidic channels in porous scaffolds can mimic the native vascular system and allow new tissue infiltration into tissue engineering scaffolds (Mao et al., 2012).

Organ-on-a-chip has been developed as a novel microfluidic cell culture model to address challenges in regenerative medicine and drug discovery. Traditional microfluidics device is commonly fabricated from polydimethylsiloxane (PDMS) due to its low toxicity, optical transparency, biological/chemical inertness, high compliance, and flexibility (Chung et al., 2012). However, PDMS-based devices are not biodegradable and are usually fabricated at elevated temperatures, which negates the incorporation of bio-functional components during device fabrication. Therefore, exploiting more biologically relevant materials is of great importance to turn microfluidics into a generic technique for broader biological applications. Silk-based hydrogel is a suitable material for microfluidic devices fabrication because of its biocompatibility and biodegradability, optical transparency, interconnected inner structure, and the ability to incorporate active biomolecules and living cells during device fabrication (Zhao et al., 2016). A biofunctionalized silk hydrogel microfluidic chip was designed and fabricated by the aqueous based multilayer fabrication method (Zhao et al., 2016). Mechanically activated valves were also integrated in the chip to demonstrate pneumatic control of microflow. This chip has highly tunable degradability and has great potential in implantable microfluidic devices. In addition to cell culture models that are made of static materials, smart culture systems that is made of stimuli-responsive biomaterials have also been developed recently as novel strategies to increase the throughput of current static culture systems (Parker et al., 2020). Recombinant silk smart carrier arrays were designed and fabricated with thermal responsive SELPs to process and analyze a large number of 3D organoid tissue cultures (Parker et al., 2020). The thermal responsive properties of SELPs allowed device contraction to secure the organoids, thus obtaining multi-sample constructs for high throughput histological analysis of organoids (Figure 7B) (Parker et al., 2020).

### Soft Robotics

Robotic systems have traditionally been made from hard materials such as metal and plastic so that they can perform tasks that are dangerous, difficult, repetitive, or inconvenient for human labor. Most rigid robots have limited applications in complex and unpredictable environments or underwater. Soft robotics, which is made of soft and/or extensible materials, have been developed in recent years to endow robots with adaptability to external environments (Kim et al., 2013). Sophisticated natural systems, such as animals and plants, have been becoming attractive inspirations for the fabrication of soft robotics (Li et al., 2020b). An actuation system is an essential component in a soft robotics system to generate reaction forces. Low-modulus materials, such as elastomers, are commonly used in actuators to sense and respond to environmental stimuli, including temperature, pressure, or chemical stimuli. By developing such intelligent multi-responsive material systems compliant with natural biological components, soft robots can be implemented into various fields, including medicine, disaster response, and human assistance (Kim et al., 2013).

**FIGURE 8:**
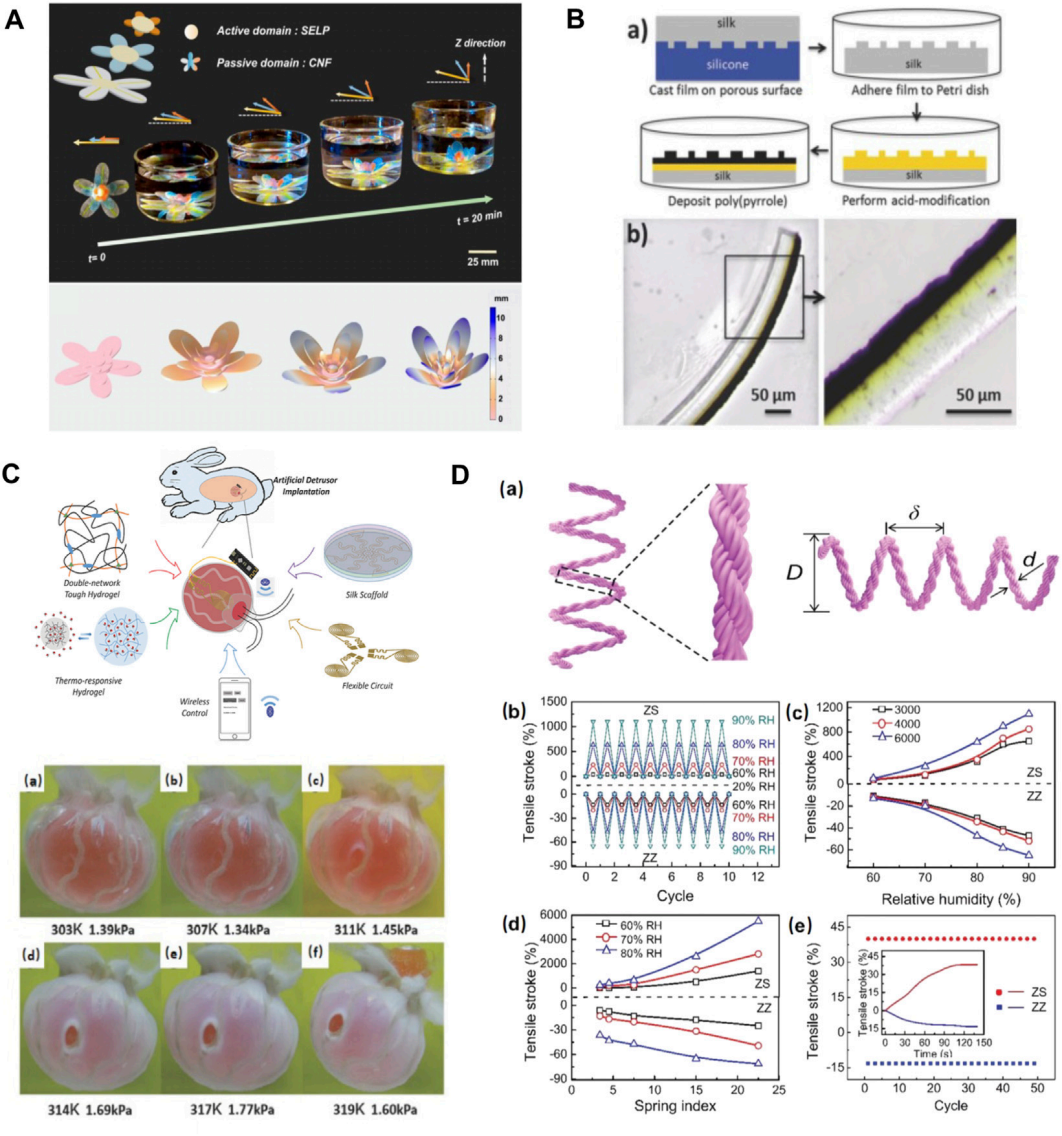
Silk-based biomaterials in soft robotics. **(A)** SELP/CNF artificial flower can alter the petal orientation. The biomimetic SELP-based multilayer artificial flower experienced a shape folding in 1 M NaCl solutions due to the response of SELP to the ionic stimulus, which is similar to the petal movements of a natural flower. Adapted with permission from ref (Wang Y. et al., 2020). Copyright 2018 Proceedings of the National Academy of Sciences. **(B)** The formation of an acid-modified silk-polypyrrole bilayer actuator (yellow part: acid modification, black part: polypyrrole). Adapted with permission from ref (Romero et al., 2014). Copyright 2014 WILEY-VCH. **(C)** Synoptic diagrams for the structure and operating principle of the artificial detrusor. Adapted with permission from ref (Yang et al., 2018). Copyright 2018 WILEY-VCH. **(D)** Fabrication and performance of tensile silk muscles. Adapted with permission from ref (Jia et al., 2019). Copyright 2019 WILEY-VCH.

Enzymatically crosslinked silk hydrogels represent an exciting new biomaterials option for *in vivo* biomedical soft robotics. These hydrogels have been traditionally used in tissue engineering (Partlow et al., 2014) due to their exceptional resilience, highly tunable properties, optical transparency, biodegradability, and biocompatibility. Recently, stimuli-responsive recombinant silk hydrogels have been rationally designed and synthesized via synergistic integration of genetic engineering and simulation (Huang et al., 2016). Inspired by the hydration-driven botanic systems, thermal and ionic strength responsive SELPs were integrated with cellulose nanofiber mat to form a bilayer actuation system (Wang Y. et al., 2020). This biocompatible actuator was able to conduct programmable deformations in response to external stimuli underwater, showing great promise in applications for *in vivo* biomedical soft robotics and bionic devices (Figure 8A) (Wang Y. et al., 2020). In addition, electromechanical multilayer actuator devices were fabricated using silk fibroin and poly (pyrrole). Polypyrrole layer exhibited volume changes due to ion/solvent diffusion in and out of the polymer matrix upon electrochemical switching between oxidized and reduced states, while the silk layer served as a static layer to control the locomotion. Chemical modification techniques were applied to produce an interpenetrating network between the silk layer and poly (pyrrole) layer to prohibit delamination (Figure 8B) (Romero et al., 2014; Li et al., 2020b). This silk-polypyrrole was stable to repeated actuation and can generate forces comparable with natural muscle, making it an ideal candidate for soft robotics application. Soft robotics devices were also applied *in vivo* to mimic the prosthesis function of human organs. For example, in fabricating a soft bladder detrusor, SF was manufactured into scaffolds that support the functional units (Figure 8C) (Yang et al., 2018). The application of the SF scaffold ensured the unification of reliable actuating ability and biocompatibility to the adjacent tissue. This silk-based artificial detrusor raised a pathbreaking possibility for bladder prosthetics by wrapping artificial detrusor around the bladder instead of complete replacement (Yang et al., 2018). Recent studies also demonstrated the application of silk-based actuators in artificial muscles, including the reversible torsional and tensile silk muscles made by self-balanced silk fibers (Figure 8D) (Jia et al., 2019) and the electrosensitive silk-polypyrrole muscles (Romero et al., 2014). These silk-based deformable materials respond to various signals in different forms of deformation, providing multiple possibilities for the design of artificial muscles.

### Biosensors

BioMEMS, a subset of microelectromechanical systems (MEMS), has emerged as a revolutionary technology with applications in biological science and biomedical engineering. Areas of research and applications in BioMEMS range from clinical diagnostics to consumable electronics, such as biosensors, implantable devices, electronic skin, wearable devices, and smart textiles (Zhu et al., 2016). The material used in MEMS technology is predominantly silicon, but BioMEMS are commonly operated in contact with tissues and organs, which raises an additional claim to the biocompatibility of the materials being used.

**FIGURE 9:**
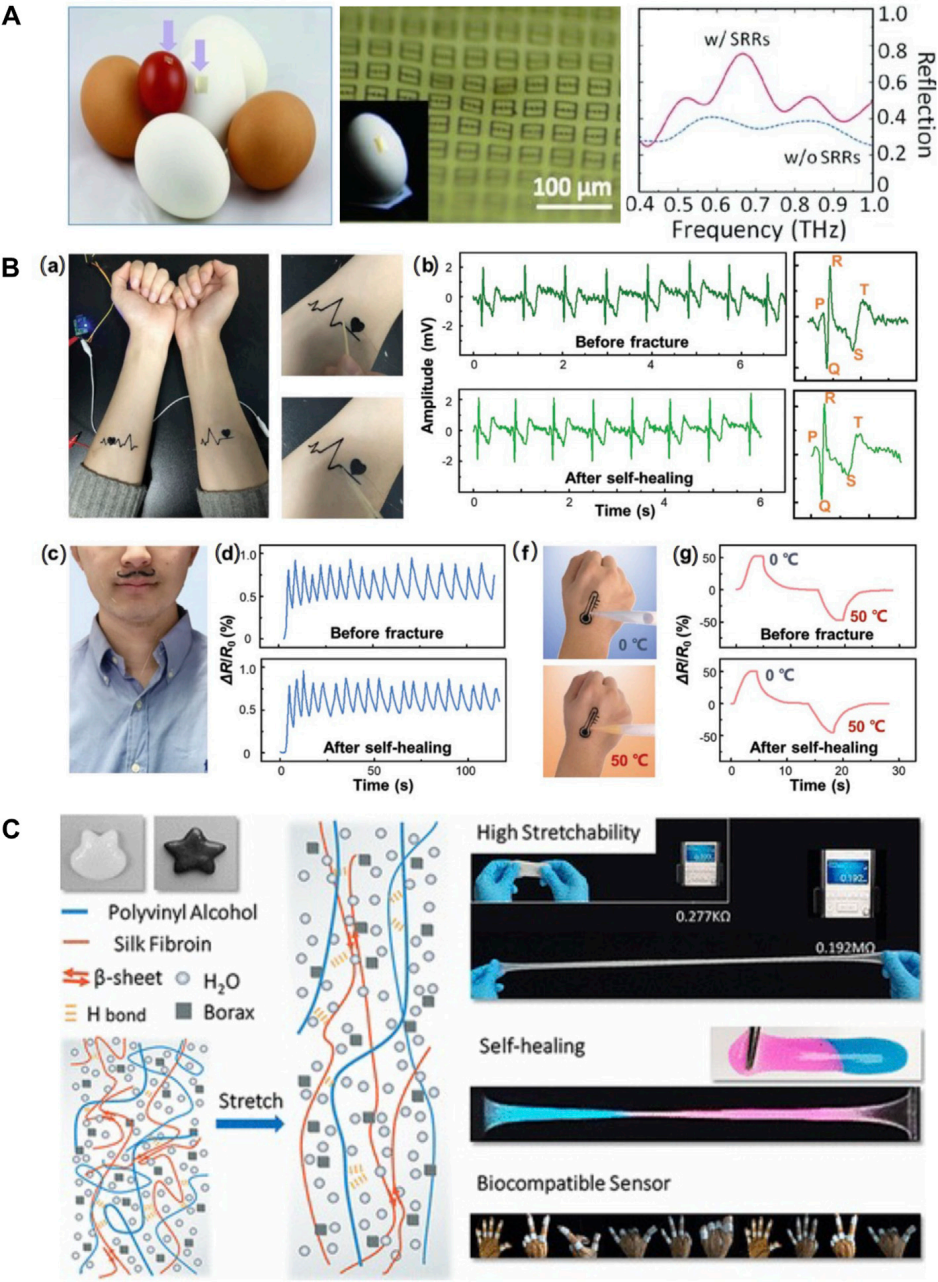
Silk-based biomaterials in biosensor. **(A)** A conformal and adhesive silk-based food sensor was fabricated by printing an array of metamaterial antennas on the silk substrate. Adapted with permission from ref (Tao et al., 2012a). Copyright 2012 WILEY-VCH. **(B)** Self-healable multifunctional E-tattoos based on graphene/silk fibroin/Ca^2+^ materials. **(A)** Flexible silk-based E-tattoos demonstrate robust attachment to the skin. The E-tattoos possess strong self-healing ability and can maintain their functions after the self-healing process for **(B)** ECG sensing, **(C)** respiration tracking, **(D)** humidity sensing, and **(F–G)** temperature sensing. Adapted with permission from ref (Wang Q. et al., 2019). Copyright 2019 American Chemical Society. **(C)** Schematic properties and possible usages of PVA/silk fibroin/borax hydrogel. The interaction of β-sheet ensured its capacity of extensive stretch. The reintegration of hydrogen bonds facilitated the self-healing of the gel. These highly stretchable and self-healing PVA/silk fibroin/borax hydrogels exhibit a promising future in biocompatible sensors. Adapted with permission from ref (Yang N. et al., 2019). Copyright 2019 WILEY-VCH.

Silk-based material offers an effective and appealing platform for the development of biocompatible and implantable flexible electronics, as they exhibit remarkable advantages in terms of low-immunogenicity, biocompatibility, and biodegradability. The robust mechanical properties, together with the insulating nature of silks, make them excellent candidates to serve as supporting and packaging materials in biosensors. For example, a conformable and adhesive silk-based food sensor was fabricated by patterning an array of passive metamaterial antennas on the SF substrates. This food sensor was able to conformally adhere to a variety of food surfaces and provide *in situ* monitoring of food quality by measuring resonant frequencies (Figure 9A) (Tao et al., 2012a). In addition, the versatile solution-processability of silks enable the integration of highly conductive materials into the silk matrix for the fabrication of conductive component in BioMEMS. Conductive nanomaterials, including graphene, carbon nanotubes, MXene, and metallic nanoparticles, as well as electronically conductive polymers, such as polypyrrole (PPy), polyaniline (PANI), and poly 3,4-ethylene-dioxythiophene (PEDOT), have been compounded with silks to construct conductive biocomposite for biosensors. For example, self-healable multifunctional E-tattoos have been printed using graphene/SF/Ca^2+^ suspension. These highly flexible E-tattoos can be intimately mounted on human skin to monitor stain, humidity, and temperature with high sensitivity, showing promising potential as epidermal electronics (Figure 9B) (Wang Q. et al., 2019). Silks can also be processed into highly elastic hydrogel under aqueous conditions to integrate mobile ions and other elastic polymers for the fabrication of hydrogel ionotronic devices. A highly stretchable hydrogel ionotronics was prepared by mixing SF with polyvinyl alcohol and borax. Due to the dynamic hydrogen bonding under wet state, the resultant polyvinyl alcohol/SF/borax hydrogel features strain larger than 5,000%, self-healing properties, and tunable conductivity, offering a new sensing platform for healthcare monitoring (Figure 9C) (Yang N. et al., 2019).

## Conclusion

Silks have been redefined and reinvented as a promising biomaterial platform for a variety of emerging applications beyond their traditional roles in textiles and sutures. In recent decades, regenerated *B. mori* silk has been functionalized for tailored chemical features and processed with nanostructures towards the next generation sustainable biodevices. With the advent of advanced genetic engineering techniques, silks have been emulated and redesigned to achieve new functions with the aid of multiscale simulation. New recombinant silks were built from the molecular level to possess both good mechanical properties from silk domains, along with desired physicochemical properties and/or biological functions from the additional functional peptides. With advances in modification and functionalization approaches, new and innovative silk material formats are being developed, including nanoparticles, micelles, nanopatterned thin films, multilayer hydrogels, and 3D micro-structured scaffolds. As a natural material that demonstrates mechanical robustness, flexibility, biodegradability, and biocompatibility, SF has been applied in the fabrication of 3D cell culture systems, soft robotics, and biosensors. 3D micro-structured SF scaffold and organ-on-chip SF device have shown great promise in regulating cell fate and tissue outcomes for tissue regeneration. Multilayer SF composites have been designed into artificial organs and soft robotics. SF film and conductive SF nanocomposite have been exploited as supporting materials and electrodes in biosensors. Stimuli-responsive recombinant silks have also been developed into smart 3D culture devices and soft actuators.

Nevertheless, there are still several challenges that need to be addressed for the fabrication of silk-based biodevices. As the SF molecular weights commonly vary from batch to batch, the enzyme-catalyzed degradation of SF-based biodevices may result in different material degradation durations (Liu et al., 2022). Hydrogel has been considered the most promising material form for regenerative medicine applications based on its porous structure, water-rich interior, and small molecule carrying capability. However, the mechanical properties of SF hydrogels limit their applications to soft tissue regeneration. Moreover, during the fabrication process, researchers use the enzymatic crosslinking method to prepare SF hydrogels, which has successfully enhanced the mechanical properties, biocompatibility, and biodegradability of SF hydrogels (Partlow et al., 2014). However, the enzymatic crosslinking process usually requires a long-period time of gelation. At the same time, materials suitable for novel technologies such as 3D printing and non-invasive treatment are expected to be capable of forming gels in a very short period, usually several seconds. Therefore, there remain challenges to seeking eco-friendly, effective, low-cost, and biocompatible means to fabricate SF-based hydrogel materials with excellent mechanical properties and well-designed functions. The *de novo* construction of recombinant silk protein materials opened new research directions that vastly broadened the application potentials of silk-based materials. Through the rational design of the polymer peptide chain, functional groups can be genetically added to the peptide, endowing silk-based recombinant proteins with new functions such as enhanced mechanical properties, light-triggered crosslinking capability (Narayan et al., 2021), and stimuli-responsiveness. However, the production of recombinant silk protein still faces the dilemma between low yield and high molecular weight, and the optimization of the protein expression process and purification process are urgently needed. Additionally, the rational design of recombinant silks, though facilitated by multiscale simulation, is still challenging at mesoscopic and macroscopic levels. Moreover, in addition to the challenges in fabricating silk biodevices, the clinical application of silk-based devices is still at a rudimentary stage. Although the silk-based tissue engineering devices and organ-on-chips have been designed to mimic living organisms, current biodevices are still in stark contrast to living tissues. Further investigations are required to develop the hybrid silk biomaterials with a more comprehensive design for clinical application. In addition, obtaining FDA approval for new silk devices and new recombinant silks are also critical to boost the future industrialization of silks.

Overall, thinking about far reaching efforts in future endeavors, two primary focuses have been established with the same goal of exploring broad applications of silks from sustainable high-performance biomaterials to biomedical related applications. One is to develop technologies to process silk into functional materials that have similar mechanical strength, function, and nanostructure to human tissues/organs on top of building a complete understanding of human body system. The other focus is to further understand the structure−function relationships and to design new recombinant silks with new functions. High-throughput screening and machine learning can be integrated with genetic engineering approach to provide a more robust and efficient strategy to address the need for new functional biomaterials. With natural and recombinant silks receiving increasing attention from interdisciplinary researchers, silk-based sustainable biodevices has shown unlimited potential to address current and future biomedical needs.
